# Megacystis-Microcolon-Intestinal Hypoperistalsis Syndrome: A Case Report

**DOI:** 10.1155/2009/282753

**Published:** 2009-09-24

**Authors:** Mehmet Melek, Yesim Edirne, Burhan Beger, Mecnun Cetin

**Affiliations:** ^1^Department of Pediatric Surgery, Yuzuncu Yil University, 65100 Van, Turkey; ^2^Department of Pediatry, Yuzuncu Yil University, 65100 Van, Turkey

## Abstract

Megacystis Microcolon Intestinal Hypoperistalsis Syndrom (MMIHS) is a quite rare congenital and fatal disease which was firstly defined by Berdon and his colleagues. It appears through a widely enlarged bladder and microcolon and its cause is unknown (Berdon et al., 1976; Carmelo et al., 2005; Makhija et al., 1999; Loening-Baucke and Kimura 1999; Redman et al., 1984; Hsu et al., 2003; Yigit et al., 1996; Srikanth et al., 1993). The disease is found in females three or four times more than in males (Srikanth et al., 1993; Sen et al., 1993; Hirato et al., 2003). Most of the cases die within the early months of their lives (Yigit et al., 1996; Srikanth et al., 1993; Sen et al., 1993; Hirato et al., 2003). We present the case of a female newborn with antenatal ultrasound revealing intestinal mass and bilateral hydroureteronephrosis. The case was admitted for intestinal obstruction after birth.

## 1. Case

A female infant with an antenatal diagnosis of intra-abdominal mass and bilateral hydronephrosis was born at a gestational age of 37 weeks through caesarean section to a grand multipar mother. After 24 hours delay of urination and meconium, she was referred to our department. Physical examination showed a distended abdomen and bladder and enlarged kidneys were palpated bilaterally. A 5 Fr urethral catheter was inserted and urine was drained. Negligible amount of meconium was seen by rectal tube insertion. Abdominal X-Ray showed a dilated stomach and minimal gas in the distal bowel segments. Abdominal ultrasound imaging revealed an enlarged urinary bladder and bilateral hydronephrosis with urethral dilatation.

**Figure 1:**
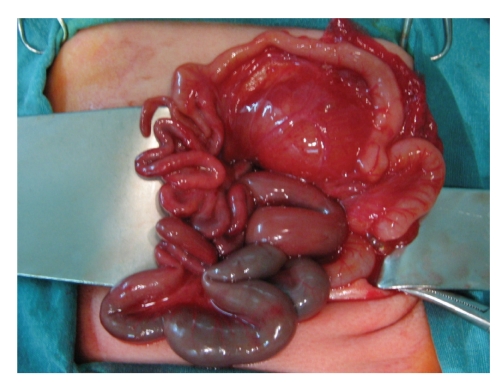
Unused microcolon with several plugs.

The case was operated for intestinal obstruction 72 hours after birth. The findings included an unused microcolon with a plug from the ileocecal valve (Figure 1). Ileotomy was made proximal to the dilated segment, and warm 0.9% NaCl was used to remove the plug without success. The narrow segment was excised, and ileocolic end-to-end anastomosis was performed.

**Figure 2:**
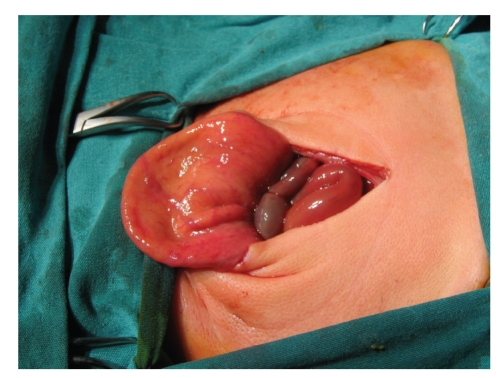
Empty megacystic bladder.

**Figure 3:**
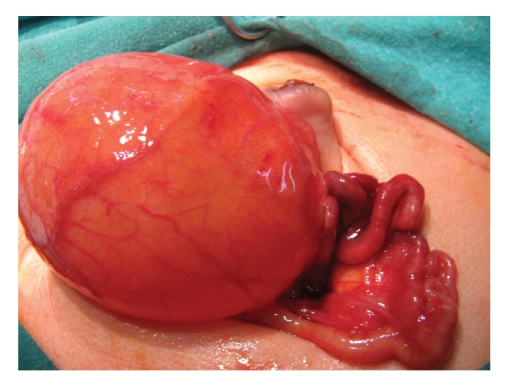
Distended megacystic bladder.

The kidneys on both sides were hydronephrotic by palpation nearly twice of the normal size, and the bladder was observed to be gigantic with megasystic property (Figures 2  and 3). The histology of the intestines showed immature Ganglion cells, a very thin muscular layer, vacuolic degeneration, and collagen proliferation. These findings confirmed our suspected diagnosis of MMIHS. Total parenteral nutrition (TPN) was started. Despite rectal tube and rectal washing no stool was observed. At five weeks the patient was referred for organ transplantation.

## 2. Discussion

Gastrointestinal dysmotility can appear as different phenotypes of an enteric neuromuscular disease globally considered as chronic intestinal pseudo-obstruction, a disorder characterized by persistent failure of the intestine to propel its contents through an unobstructed lumen. Chronic intestinal pseudoobstruction may be primary, as in most pediatric cases, or secondary to various disorders (muscular dystrophy, connective tissue diseases, chronic infections such as Chagas disease, etc.) that usually occur in adults. The congenital conditions are associated with extraintestinal symptoms. Eighty-five percent of patients with myopathy and 10% with neuropathy have megacystis (dilated bladder with altered function) [1]. 

MMIHS, which is a rare reason of the neonatal intestinal obstruction, is a congenital disease with high mortality rates [1, 2]. It is characterized with hypoperistalsis or aperistalsis of gastrointestinal system, nonobstructive bladder distension, malrotation, dilate proximal ileum, and colon [1, 3, 4]. 

MMIHS is part of the spectrum of intestinal motility defects [1]. Autosomal recessive transition has been defined in most publications which report family history and sibling cases [5–9]. The frequency of the disease is observed three or four times more in girls than in boys [4, 6, 10].

Our case was a female infant who was the 8th life birth of a 37-year-old mother. The parents were first degree relatives.

A consensus on the theory of the pathogenesis of the disease does not exist yet. Some of the theories are the lack of nicotinic acid receptor subunits, a defect in fiber synthesis, an inflammatory process of the gastrointestinal and urinary tract, generalized axonal dystrophy in central, and peripheral and autonomic nervous system [3, 8, 9, 11–15].

Normal or excessive amounts of ganglion cells have been reported. Also, a case without ganglion cells has been reported, too [2, 3, 11, 12, 16, 17]. In addition, vacuolization and degeneration in bladder and intestinal smooth muscle have been shown in pathological examinations [8, 12, 18]. 

The ganglion cells obtained from the narrow distal ileal segment were mostly immature but positive and in normal number according to pathological examination result. The immaturity in our case is supporting the theory about cell defect.

Although there has been publications about maternal drug usage for defining the etiology, there is an emphasize on the necessity of advanced experimental studies [7, 8]. We learnt that the mother in our case had used analgesics during her pregnancy.

The antenatal diagnosis of the MMIHS is very difficult. However, publications about antenatal symptoms and additional urinary system anomalies were reported [5–7, 13]. Although Ultrasonography (USG) is a valuable method for diagnosis, it is insufficient in evaluating the viability of the intestinal tract and microcolon [5–7, 19]. Imagination of an enlarged bladder with a microcolon is necessary for a diagnosis by MRI [19]. 

Our case was admitted to our clinic because of mass in the abdomen due to globe vesicale and bilateral Grade III-IV hydronephrosis in antenatal and postnatal USG and was hospitalized. 

Surgical treatment choices are limited, and an effective surgical method has not been defined yet. Several intestinal diversions have failed to be successful [6, 10, 16]. Despite common use in many cases, prokinetic agents are not found to be effective on peristalsis [6, 8, 12, 16, 17]. 

These cases mostly die from malnutrition, sepsis, kidney failure, and liver failure depending on TPN and the complications of TPN. [1, 2, 6, 10, 12, 17, 20] Multiorgan transplantation is suggested as a valuable alternative for children with severe gastrointestinal dismotility. [1, 4, 8, 21] Treatment is supportive and involves an ileostomy to defunction the colon, with TPN. We applied narrow distal ileum resection and ileocolic anaostomosis in this case, and TPN was continued for 5 weeks. Oral feeding was impossible and ileocolic anastomosis also failed to pass feces. The case was referred for organ transplantation. Our case died of sepsis after 3 weeks at the transplantation center. 

## 3. Result

Prenatal diagnosis of MMIHS is possible by antenatal ultrasound. Studies of the contribution of MRI in the prenatal evaluation of digestive tract anomalies are still rare. MRI also appears useful for diagnosing microcolon associated with an enlarged bladder, suggesting MMIHS [19].

In our case, antenatal USG revealed intraabdominal mass and bilateral hydronephrosis at 32 weeks and resulted with a live birth at 34 weeks.

We suggest that an antenatal USG finding of an enlarged urinary bladder and intraabdominal mass in female fetus should alert the physicians for MMIHS. Clinical genetic counseling is indicated for further pregnancies.
